# Moxibustion for primary dysmenorrhea: A protocol for evidence-based clinical practice guideline

**DOI:** 10.1097/MD.0000000000024466

**Published:** 2021-02-19

**Authors:** Rongrong Nie, Shouqiang Huang, Weiye Liao, Zhiming Mao, Xianglin Li, Jun Xiong

**Affiliations:** a Rehabilitation treatment department of Affiliated Hospital of Guilin Medical University, Guilin; b Jiangxi University of Traditional Chinese Medicine; c The Affiliated Hospital of Jiangxi University of Traditional Chinese Medicine, Nanchang, China.

**Keywords:** clinical practice guidelines, moxibustion, primary dysmenorrhea, protocol

## Abstract

**Background:**

Primary dysmenorrhea (PD) is a common gynecological disease characterized by lower abdominal pain. Moxibustion as a traditional Chinese treatment, can effectively treat PD with few adverse reactions. Nowadays, there is still no standard guideline for moxibustion treatment of PD, so related clinical practice guidelines need to be developed.

**Methods:**

This guideline will be developed in line with the latest guideline definition from Institute of Medicine, and that applies the GRADE system as well as the World Health Organization handbook to appraise the quality of evidence and develop recommendations. We will set up a Guideline working group, put forward the corresponding problems based on the principle of Population, Intervention, Comparison, Outcomes (PICO), and complete the literature retrieval. After achieving consensus through evidence syntheses and 2 to 3 rounds of Delphi process, we will also consider patients values and preferences and implement peer review in the guideline.

**Result:**

We will put forward evidence-based best practice recommendations and moxibustion standard to improve the symptoms caused by primary dysmenorrhea in a more efficient way. At present, the research is still in progress, and there is no result to report.

**Conclusions:**

This guideline will be helpful to clinical acupuncturists and other professionals to further improve clinical efficacy in treating PD with moxibustion. Moreover, we will also constantly update and evaluate the evidence to both support recommendations and identify gap areas for future research.

**Systematic Review registrations:**

registration number: IPGRP-2020CN021

## 1 Introduction

Primary dysmenorrhea (PD), also known as functional dysmenorrhea, that is the menstrual cramps with no apparent organic lesions. Its typical symptoms are characteristic by crampy, colicky spams of pain during menstruation, and regularly coupling with dizziness, sickness, emesis, diarrhoea, and fatigue, even cold limbs, syncope and other severe symptoms.^[1]^ In some case, this situation bring much pain to the patients.

PD are common affliction among women, whose prevalence is relatively high among adolescent and young adult females. The previous study has reported that the PD prevalence in global women was 25%, while was 90% in global teenagers and approaching 15% of patients feeling in great pain.^[2,3]^ A recent study in Japan has shown that the morbidity of dysmenorrhea varies with age, wherein 12 years old, its ratio was 31.6%, 13 years old was 39.5%, 14 years old was 50.3%.^[4]^ In China, the morbidity of dysmenorrhea accounts for 33.1%, that more than half is primary dysmenorrhea, greatly affecting patients both physically and mentally.^[5]^

The reason for PD is related to many factors, and the pathogenesis not yet fully elucidated. It has been proved that PD was associated with the hypercontractility of uterine smooth muscle and spiral artery of uterine wall, so that inducing ischemia and hypoxia and triggering pain in the hypogastrium.^[6,7]^ Recently years, many studies have defined the over synthesis of prostaglandin in the endometrium as the dominant reason for PD, especially the PGF2α and PGF2 that play a fundamental role in increasing uterine muscle tone and high-intensity contraction, triggering the acid metabolites adding up in the myometrium thus causing pain.^[8–11]^ Otherwise, some studies suggests that ET and NO are also important factors causing primary dysmenorrhea, those mainly regulates uterine vascular tone and blood flow.^[12]^

For the treatment of PD, the currently recommended pharmacological therapies of PD mainly included nonsteroidal anti-inflammatory drugs, prostaglandin antagonists, oral contraceptives and anti-spasmodic drugs. To some extent, these medications can temporarily alleviate pain, but it is associated with some shortcoming, such as high recurrence rate, drug resistance, particularly when used for the long term, which is easy to produce gastrointestinal discomforts, and long haul renal dysfunction.^[13,14]^ Therefore, seeking complementary and alternative treatment for PD to relieve pain is significant. Traditional Chinese medicine described that primary dysmenorrhea is closely related to “stasis,” “stagnation” and “deficiency,” and should be treated by the principle of warming yang for dispelling cold, activating blood circulation, and unblocking collaterals.^[15]^

Moxibustion, as an external therapy of traditional Chinese Medicine, has a wide range of applications to prevent and treat diseases with the advantages of simple operation and good economic benefits. As we all know, the warm and hot stimulation of moxibustion can warm the meridians and activate the collaterals and keep the Qi and blood running unobstructed to relieve the pain symptoms. Modern studies have confirmed that moxibustion can redistribute microcirculatory blood flow in the body by affecting the function of microvascular relaxation and contraction.^[16]^ Another study has shown that moxibustion can significantly reduce the levels of PGF2ɑ and PGF2ɑ/PGE2 in uterine tissues of Dysmenorrhea Rats, and improve the activity of NK cells in the spleen of rats.^[17,18]^

Although plenty of studies have reported the curative effect and safety of moxibustion for PD, there is still a lack of guidelines involving moxibustion for PD, so we need to formulate relevant clinical practice guideline (CPG). Moreover we preliminary found more than 200 RCTs as well as some SRs or meta-analysis using moxibustion to treat PD, all that have been regarded as the basis of drawing up CPG.

## 2 Aims

The aim of this study is to formulating systematic guidelines and moxibustion standard that can be used by acupuncture practices as well as other specialities to further improve the management of primary dysmenorrhea with moxibustion.

## 3 Method

### 3.1 Principle

Regarding guideline development, we closely comply with the Institute of Medicine^[19]^ as well as the World Health Organization handbook for guideline.^[20]^ In addition, we also follow 6 domains of the AGREE II instrument^[21]^ and GRADE system.^[22]^

### 3.2 Study registration

We have registered the guideline on the International Practice Guidelines Registry Platform (http://www.guidelines-registry.org/guid/875), and the registration number is IPGRP-2020CN021.

### 3.3 Participating institutions

The CPG started in February 2020 and was launched by Affiliated Hospital of Jiangxi University of Traditional Chinese Medicine, which will be titled, ‘Moxibustion for primary dysmenorrhea: evidence-Based Clinical Practice Guideline.’

### 3.4 End-users and target population

The guideline's target users including acupuncturists, physicians, journal editors as well as other relevant researchers. And the patients treated with moxibustion compose the target population.

### 3.5 Guideline working group

The Guideline working Group consisted of the Guideline Development Group, the Guideline Steering Group and the Guideline Secretary Group, will be established in March 2020. To ensure the process fair and scientific, various tasks is conducted by experts in each group from several fields of specialisation. The team members and tasks are as follows:

The Guideline Development Group will be composed of 12 acupuncturists (with rich clinical experience of PD), 3 TCM physicians, 3 physiotherapists, 2 medical clinicians, an editor, a health economist physician and a nurse; and they will be in charge of the following mission:

(1) to develop the scope of the guideline, the population of the draft, Intervention, Comparison, Outcomes (PICOs);
(2) to assess the evidence quality;
(3) to make initial proposal;
(4) to formulate a draft guideline;
(5) to publish and generalize the guideline.

The Guideline Steering Group will be composed of 3 acupuncturists, 1 evidence-based medical experts, 1 TCM physician, 2 physiotherapists and 1 health economist physician; and they will be in charge of the following mission:

(1) to authorize the guideline's scope and PICOs;
(2) to supervise literature search and systematic reviews;
(3) to examine the quality of evidence;
(4) to develop the final recommendation based on the revised Delphi approach;
(5) to ratify the release of the guidelines.

Guideline Secretary Group: The member of the Guideline Secretary Group will be composed of 3 evidence-based medical experts, 2 acupuncturists and 1 statistician. and they will be in charge of the following mission:

(1) to do a literature research and accomplish systematic reviews;
(2) to survey patients’ viewpoint and favor.
(3) to assist the development of the guideline.

### 3.6 Statement of interest

To ascertain their underlying benefit conflict, all individuals engaged in the Guideline Working Group should accomplish the s*tatement of Interest* forms to enhance the transparency and credibility of the CPG.

### 3.7 Identifying the problem and selecting the outcomes

We will finalize the PICOs after the scope of guideline defined by the Guideline Steering Group and examined by the Guideline Steering Group. The Guideline Development Group will evaluate the importance of the outcomes. The outcomes will be categorized as critical, important, not important by the score of 1 to 9. wherein, 7 to 9 will be regarded as critical to make a decision and develop recommendations, 4–6 as important and 1 to 3 as not important. According to PICOs principle, we will throw out the recommendations from the aspects of safety and effectiveness of moxibustion, moxibustion method, moxibustion doses as follow.

Is moxibustion effective and safe for PD?

P: Patients diagnosed with PD
I: Patients who treat with moxibustion
C: Patients who treat with other therapy
O: The Primary outcome is the overall response rate for PD treated with moxibustion. The secondary outcomes mainly including Cox Menstrual Symptom Scale, frequency of analgesic use, and visual analog scale scores. The moxibustion-related adverse events are recorded in the case report form, such as scalding, allergy, pregnancy, skin vesicles, skin pain and others, all that is documented throughout the trial.

How to implement moxibustion normatively and effectively?

P: Patients diagnosed with PD
I: Patients who treat by moxibustion with dynamic operation
C: Patients who treat by moxibustion in fixed point without dynamic operation
O: The total effective rate, rate of adverse events and the level of plasma PGF2a.
How to select the optimal amount of moxibustion for each patient?
P: Patients diagnosed with PD
I: Patients who treat by moxibustion with individualised ‘sensitivity elimination’ dose^[23]^
C: Patients who treat by moxibustion with a standardised 15 min dose
O: The total effective rate, the Cox Menstrual Symptom Scale and the visual analog scale scores.

### 3.8 Retrieving evidence

We will systematically search the literature until June 30, 2020, including three foreign Databases- Embase, PubMed, Cochrane library, and four Chinese literature databases- CNKI, SinoMed, Wanfang and VIP. Free words and MeSH terms will be searched synchronously with the help of evidence-based experts. Such as primary dysmenorrhea, essential dysmenorrhea, functional dysmenorrhea, moxibustion, indirect moxibustion, suspended moxibustion, direct moxibustion, mild moxibustion, heat-sensitive moxibustion. There will be no restrictions on publication language, and references must have clear diagnostic and inclusion criteria.

### 3.9 Choosing literature

Firstly, we will eliminate some unqualified studies according to the title and abstract, then bring into relevant literature by reading the whole text, such as systematic review, meta-analysis and original studies. Meanwhile, we plan to conduct a pre-test before the literature selection that could ensure the consistency of literature choice criterion.

### 3.10 Evidence syntheses

The high-quality systematic reviews complying with PRISMA guidelines released in recent two years will be adopted directly. If we get low-quality systematic reviews, we will apply the currently available evidence to conduct new systematic reviews.

### 3.11 Evaluating evidence

The assessment of evidence quality will be divided into high, moderate, low or very low with the GRADE instrument. Such as the randomised controlled trials are considered as high-quality, and observational studies as low-quality. In addition, three escalation factors (large effect size, confounding factor bias, and dose- effect) and 5 downgrading factors (ask of bias, inconsistency, accuracy, publication bias and indirection) will be considered. The guideline methodologists will be in charge of evaluating the evidence quality, drafting the evidence summaries, and then submitting to the Guideline Working Group.

### 3.12 Patient's values and favours

We will survey the value and preference of moxibustion treatment for PD, which mainly includes the effectiveness of moxibustion therapy for AR, adverse reactions, burdens, costs, potential benefits, and so on. Meanwhile, the reliability and acceptability of the findings will be evaluated to inform the development of the clinical questions. The finding will be referenced by the experts of the guideline working group when formulating the recommendations. Besides, the patients will be required to receive related training and sign the informed consent before the investigation.

### 3.13 Developing recommendations

According to the evidence quality, the values and favours of patients, the balance between the pros and cons, and the economic analysis, the preliminary recommendation will be drafted by the Guideline Development Group. With 2 to 4 rounds of the Delphi process, the draft recommendations will be submitted to he Guideline Steering Group for final approval. We will vote by GRADE grid^[24]^ to reach decisions, and the strength of recommendation is divide into strong recommendation, weak recommendation, unclear recommendation, weak no recommendation, strong no recommendation. The recommendation will be adopted on that condition the approval ratio of the experts is more than 50% for any option or more than 70% for one of the two options on same side.

### 3.14 Peer review

The guideline will be reviewed by external peer experts to ensure uniformity. The Guideline Development Group will be responsible for recording the review process, then the feedback will be discussed by experts to make the final decision.

### 3.15 Release and update of the guidelines

It is expected that the full text consistent with the requirements of RIGHT will be published in 2022. The publication will be published in relevant journals and updated it every 3 to 5 years.

### 3.16 popularizing, conducting and assessment of the guideline

After releasing the guideline, the dissemination and promotion of the guideline will be implemented by Affiliated Hospital of Jiangxi University of Traditional Chinese Medicine and Guilin Medical University. They will be in charge of the tasks as follow:

(1) The guideline will be proposed in relevant seminars and forums;
(2) A nationwide study lecture related to the guideline will be held for acupuncturists, physiotherapists, nurses and others related personnel;
(3) The experts of the guidelines will write articles regarding the guidelines and publish them in journals, popular medical or official health websites;
(4) An extensive investigation relating to the application and dissemination of the guidelines will be produced.
(5) To conduct an assessment for the implementation of the guideline.

## 4 Result

The study has not been accomplished, and there is a lack of complete data.

## 5 Discussion

In line with the recognized methodology for guideline developments, this would be the first CPG for management of primary dysmenorrhea with moxibustion, which is expected to be implemented more widely and standardly than previously, thus improving the effectiveness, safety of PD therapy. However, the above protocol still has limitation, we should recognise that the differences of moxibustion methods utilised by different country in literature retrieval.

## 6 Conclusion

In this guideline, we will strickly follow the Institute of Medicine new guideline definition and the methodology for guideline developments in hopes that achieve better results in treating PD with moxibustion. Important, the guideline will provide a set of implementation standards for moxibustion based on the rich experience of heat-sensitive moxibustion in our hospital.

## Author contributions

This protocol was drafted by Rongrong Nie. Shouqiang Huang, Rongrong Nie, Jun Xiong were responsible for the conception of the study. And this protocol was revised by Jun Xiong and Shouqiang Huang. Weiye Liao, Zhiming Mao, Xianglin Li played an important role in protocol development. All authors gave final approval for the version to be published.

**Data curation:** Zhiming Mao, Weiye Liao, Xianglin Li.

**Resources:** Zhiming Mao, Weiye Liao, Xianglin Li.

**Supervision:** Shouqiang Huang, Jun Xiong.

**Writing – original draft:** Rongrong Nie.

**Writing – review & editing:** Rongrong Nie, Shouqiang Huang.
